# Systemic Inflammation Aggravates Retinal Ganglion Cell Vulnerability to Optic Nerve Trauma in Adult Rats

**DOI:** 10.3390/ijms27031502

**Published:** 2026-02-03

**Authors:** Giuseppe Rovere, Yolanda Caja-Matas, Beatriz Vidal-Villegas, José M. Bernal-Garro, Paloma Sobrado-Calvo, Manuel Salinas-Navarro, Carlo Nucci, María Paz Villegas-Pérez, Manuel Vidal-Sanz, Marta Agudo-Barriuso, Francisco M. Nadal-Nicolás

**Affiliations:** 1 Department of Ophthalmology, Faculty of Medicine, University of Murcia and Biomedical Research Institute of Murcia (IMIB-Pascual Parrilla), 30120 Murcia, Spain; rovere292@hotmail.com (G.R.); cajayolanda@gmail.com (Y.C.-M.); jmbg@um.es (J.M.B.-G.); sobrado@um.es (P.S.-C.); manuel.salinas@um.es (M.S.-N.); mpville@um.es (M.P.V.-P.); manuel.vidal@um.es (M.V.-S.); martabar@um.es (M.A.-B.); 2 Department of Clinical Science and Translational Medicine, Section of Orthopaedics and Traumatology, University of Rome Tor Vergata, 00133 Rome, Italy; 3 Policlinico Tor Vergata University Hospital, 00133 Rome, Italy; 4 Hospital General Universitario Santa Lucía, 30202 Cartagena, Spain; 5 Department of Immunology, Ophthalmology and ENT, Faculty of Medicine, Complutense University of Madrid, 28040 Madrid, Spain; beatrizvidalvillegas@gmail.com; 6 Ophthalmology Unit, Department of Experimental Medicine, University of Rome Tor Vergata, 00133 Rome, Italy; nucci@med.uniroma2.it

**Keywords:** traumatic injury, optic nerve crush, inflammation, neuroinflammation, systemic infection, lipopolysaccharide, adult albino female rat, microglial cells, microglia activation, retina

## Abstract

Systemic inflammation is increasingly recognized as a modifier of neurodegenerative outcomes in the central nervous system; however, its impact on retinal ganglion cell (RGC) survival and retinal microglial responses following optic nerve (ON) injury in vivo remains incompletely understood. In this study, we investigated how systemic lipopolysaccharide (LPS)-induced inflammation influences retinal microglial activation and RGC vulnerability under physiological conditions and after traumatic ON damage. In adult female rats, systemic LPS administration by intraperitoneal injection induced rapid and robust microglial activation, characterized by process retraction and soma hypertrophy within hours and promoting microglial proliferation at later stages but without causing RGC loss in intact retinas. Following ON crush, systemic inflammation did not affect early RGC degeneration but significantly exacerbated neuronal loss during the late acute phase. This increased vulnerability was accompanied by a marked rise in microglial density and a pronounced redistribution of microglia toward the central retina and the ON head, a region of heightened anatomical and metabolic susceptibility. Together, these findings demonstrate that, in rats, systemic inflammation alone is insufficient to induce RGC degeneration but acts as a potent priming factor that amplifies neurodegeneration in the context of axonal injury. The temporal and spatial specificity of microglial responses underscores their context-dependent role in retinal pathology and identifies systemic inflammatory status as a critical determinant of retinal outcome after trauma. Targeted, time-dependent modulation of microglial activation may therefore represent a promising therapeutic strategy for optic neuropathies.

## 1. Introduction

Neuroinflammation has emerged as a central player in the progression of neurodegenerative diseases and in modulating the central nervous system (CNS) response to injury [1]. Microglia, the resident immune cells of the CNS [2], are critical for maintaining homeostasis, constantly monitoring and pruning synapses to maintain the health of neuronal networks while supporting neuronal function under physiological conditions [3,4]. However, under pathological or systemic inflammatory conditions, microglia can become activated [5,6], adopting a pro-inflammatory phenotype [7]. Once activated, M1 microglia adopt an amoeboid morphology, increase in number, and release proinflammatory and neurotoxic factors that modulate neuronal activity and function [8]. Activated microglial cells (MCs) also act as macrophages by phagocyting dead bodies and cellular debris [9,10,11]. However, despite this characteristic M1/M2 dichotomy, single cell sequencing analysis demonstrates that this activated state is functionally more complex [12].

The retina provides an accessible model to study CNS neuroinflammation, as it harbors all major glial types (microglia, Müller cells, and astrocytes) and exhibits well-defined neuronal circuitry. Retinal ganglion cells (RGCs), the only brain-projecting neurons that form the optic nerve (ON), are particularly vulnerable to metabolic, inflammatory and mechanic insults [13,14,15,16]. Following traumatic ON injury, RGCs and their axons undergo rapid degeneration [17,18,19,20,21,22,23] that progress slowly during the following months and years [24]. Significantly, more than 85% of RGCs degenerate during the first two weeks due to their own limited regenerative potential [25], but also to a subsequent glial reactivity, where microglia activation, proliferation and migration contribute to the clearance of moribund RGC bodies and to repair the local damage [9,11]. While ON trauma reliably activates retinal microglia and astrocytes, microglia are pivotal mediators of neuroinflammation and can induce or modulate a broad spectrum of cellular responses [26,27]. However, depletion of microglia has been proven not to improve RGC survival after ON crush (ONC) [28]. Thus, the impact of preexisting systemic inflammation on the magnitude or character of this glial response remains poorly understood.

Lipopolysaccharide (LPS), a potent endotoxin derived from Gram-negative bacterial cell walls [29], is widely used to model systemic inflammation through activation of Toll-like receptor 4 (TLR4, [30,31]). Systemic LPS administration triggers the release of proinflammatory cytokines such as TNF, IL-1β, and IL-6, which can communicate with the CNS via humoral and neural pathways and compromise blood–brain and blood–retinal barrier integrity [32,33,34,35] although other studies suggest that the neuroinflammation could be independent of these proinflammatory factors [36]. In the brain, LPS induces learning and memory deficits without neuronal cell death as consequence of microglial activation [37]; while other study reports loss of dopaminergic cells in the mouse brain [38]. In the mouse retina, intraperitoneal (i.p.) injection of LPS induces similar mechanisms with robust microglial activation, an increase of cytokine release and neuronal loss [39]. These observations suggest that systemic inflammation could induce a chronic proinflammatory microglial cell response that can persist activated even when the stimulus has dissipated [38]. Consistent with these experimental findings, sepsis in humans remains associated with high mortality and long-term neurological morbidity, with approximately one-third of patients dying within the following year and nearly one-sixth of survivors developing severe and persistent neurological impairments [40,41,42,43].

However, in pathological conditions, such as Alzheimer Disease, LPS administration has been reported to protect dopaminergic neurons after the infiltrating T cells downregulate the microglia-mediated inflammation [44]. The concept of microglial in pathological conditions in addition to subsequent insults or additional stressors provides a mechanistic framework to understand how peripheral immune activation may influence CNS resilience or vulnerability in neurodegenerative disorders. Importantly, this interaction has clinical relevance, as chronic systemic inflammation or infection is increasingly recognized as a risk factor for neurodegenerative diseases such as optic neuropathies [45,46]. Despite some inflammatory responses are beneficial for clearing debris, prolonged or uncontrolled inflammation can produce neurotoxic factors that accelerate neurodegeneration.

Here, we investigated how systemic-induced inflammation, triggered by i.p. LPS injection, affects retinal microglial activation and RGC survival after traumatic ON injury. We hypothesized that LPS-induced systemic inflammation activates retinal microglia, resulting in an exaggerated inflammatory response that may induce neuronal loss in intact retinas and/or aggravate neuronal loss following mechanical injury. In the retina, LPS exposure has been associated with diverse pathological outcomes, including retinal degeneration [47,48,49], retinal dysfunction [50], alterations in vascular development [51], and exacerbation of neovascularization in models of laser-induced choroidal neovascularization [52]. With respect to neuronal survival, LPS has been reported to induce RGC loss in male mice [39], accelerate photoreceptor degeneration in rat models of retinitis pigmentosa [53], and cause demyelination, axonal loss, and RGC degeneration when administered locally into the ON [54]. In contrast, other studies have reported neuroprotective effects of LPS against light-induced retinal stress [55], suggesting that the retinal outcome of LPS exposure may depend on dose, route of administration, sex, and the underlying pathological context.

Consistent with this context-dependent framework, our results demonstrate that in female rats systemic LPS administration (1 mg/kg in sterile saline) activates retinal microglia without inducing RGC degeneration per se but significantly exacerbates neuronal loss following optic nerve injury.

## 2. Results

We analyzed the temporal aspects of RGC death, microglial activation and cumulative profile on healthy rat retinas and after ON injury as consequence of the lipopolysaccharide (LPS)-induced inflammation by comparing microglial morphology and density, and RGC survival in adult rats subjected to a single LPS administration.

### 2.1. LPS-Induced Systemic Inflammation Triggers Microglial Cell Activation in the Retina Within Hours

**Figure 1:**
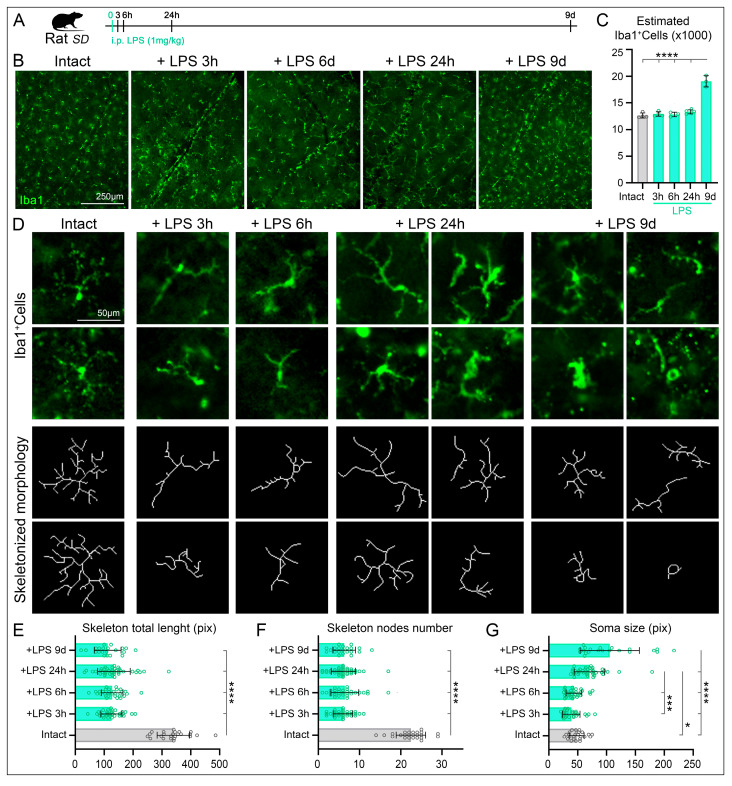
Schemes Microglial cell morphology is altered by lipopolysaccharide (LPS) administration. (**A**) Experimental design, the effects of intraperitoneal (i.p.) administration in the microglial cells after different times post-administration (3, 6, 24 h or 9 days). (**B**) Immunodetection of Iba1 in intact retinas or at different times post-administration of LPS. (**C**) Quantitative estimation of total Iba1^+^Cells after different times post-administration of LPS. Microglial cell numbers significative increase at 9 days after LPS administration (sample sizes: Intact, n = 4; LPS 3 h, n = 4; LPS 6 h, n = 3; LPS 24 h, n = 5; LPS 9 days, n = 3). (**D**) Top rows, Representative morphologies of microglial cells immunodetected with Iba1 in intact retinas or after different times post-administration of LPS. The bottom rows show the skeletonized morphologies (1 pix wide) reconstructed from the corresponding Microglial cells. (**E**) Bar graphs showing significant reduction of total microglial cell length in all different times post-LPS administration compared to intact retinas. (**F**) Bar graphs showing significant reduction of the number of nodes at different times post-LPS administration compared to intact retinas. (**G**) Bar graphs showing significant increase of the soma size of microglial cells at 24 h and 9 days post-LPS administration. Interestingly a slight reduction was observed at 3 and 6 h post LPS-administration. Data are presented as mean ± SD, with statistical significance assessed using one-way ANOVA with Tukey’s post hoc test for multiple comparisons. Statistical significance is indicated as follows: *p* < 0.05 (*), *p* < 0.001 (***), and *p* < 0.0001 (****). LPS, lipopolysaccharide.

To assess whether systemic inflammation by intraperitoneal inoculation of exogenous LPS induces a local immune response in the retina, we evaluated the density and morphology of MCs in intact retinas and at 3, 6, and 24 h, as well as 9 days after LPS inoculation, using Iba1 immunolabeling (Figure 1A,B). In intact retinas, MCs show equidistant cell bodies distribution (Figure 1B) and the average total number of microglial cells, estimated from three regions per quadrant (12 images per retina) and then multiplying it by their corresponding retinal area, was 12,641 ± 473 MCs (Figure 1C, Table 1). Identical quantitative analysis in retinas at 3, 6, or 24 h following LPS administration revealed no significant changes in microglial cell density (Figure 1C, Table 1). However, a significant increase in microglial cell number was detected at 9 days post-LPS injection 19,071 ± 1051 MCs (Figure 1C, Table 1).

Regarding microglial morphology, MCs in intact retinas displayed a resting phenotype characterized by small cell bodies and a highly ramified morphology with multiple fine processes (Figure 1D, first column). Notably, despite the absence of changes in microglial cell number at early time points, marked morphological activation was observed as early as 3 h after LPS inoculation. At this time point, MCs shifted toward an activated state, adopting a fusiform morphology, retracting most of their processes (Figure 1B–D, second column). This activated morphology persisted at 6 and 24 h post-LPS administration (Figure 1B–D, middle columns). At 9 days after LPS inoculation, microglial cells remained hypertrophic with wider processes, indicating sustained activation, consistent with a chronic inflammatory state (Figure 1B–D, right columns). These morphological changes were further corroborated by quantitative skeleton analysis. The total process length of skeletonized microglia was reduced by more than 60% compared to intact retinas (Figure 1E, Table 1), and the number of nodes was similarly decreased (more than 70%), reflecting a marked reduction in branching complexity (Figure 1F, Table 1). Soma size analysis revealed a progressive increase following LPS administration, reaching statistical significance at 24 h post-inoculation. At 9 days post-LPS, microglia exhibited the most pronounced activation parameters (Figure 1G, Table 1). Interestingly, although not statistically significant, a transient reduction in soma size was observed at 3 and 6 h post-LPS injection, suggesting a dynamic early morphological response to systemic inflammation (Figure 1G, Table 1).

### 2.2. Activated Microglial Cells by LPS Inoculation Do Not Cause Retinal Ganglion Cell Death

**Figure 2:**
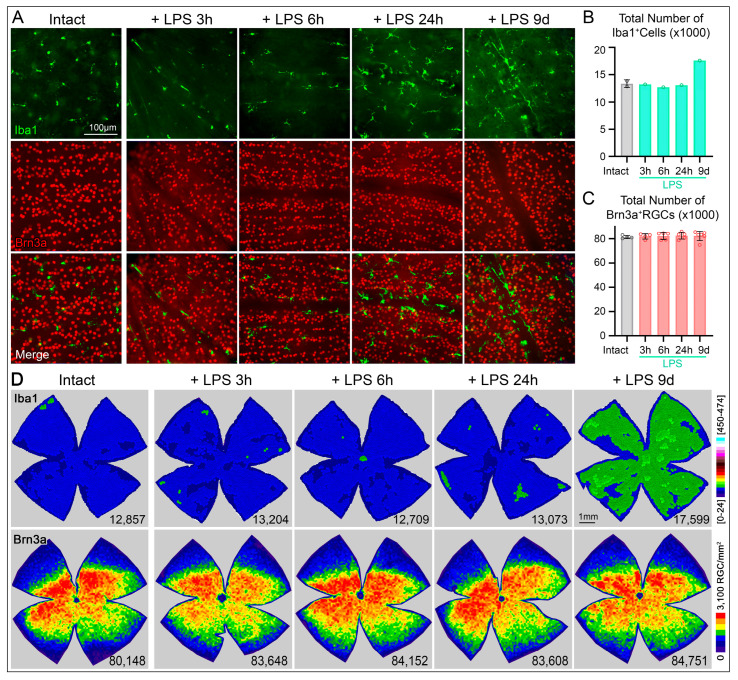
Induced in vivo retinal microglia activation by intravitreal injection of lipopolysaccharide in control retinas does not prompt RGC loss. (**A**) Representative images of microglial cells (Iba1^+^, green) and retinal ganglion cells (RGCs, Brn3a^+^, red) in intact retinas or after different times post-administration of LPS. (**B**) Bar graphs showing significantly higher total number of Iba1^+^cells in whole retinas (n = 1 retina/group; except to Intact group, n = 2) confirm the estimated number obtained by regional quantification (Figure 1B). (**C**) Bar graphs showing the total number of Brn3a^+^RGCS without significant differences among the groups studied after LPS administration compared to the intact group (sample sizes: Intact, n = 4; LPS 3 h, n = 4; LPS 6 h, n = 5; LPS 24 h, n = 5; LPS 9d, n = 6). (**D**) k-nearest neighbor maps show the topographical distribution of Iba1^+^cells (top row) and Brn3a^+^RGCs (bottom row) in rat retinas. The retina at 9d post PLS administration exhibit higher Iba1^+^cell density (evident in the K-nearest neighbor map), while RGC densities remain constant among groups. Color scales (left) indicate local density ranging from [0–24 neighbors/cell] (purple) to [450–474 neighbors/cell] (red) or (cyan) within a 345 µm radius. The bottom right of each map indicates the total number of Iba1^+^cells or Brn3a^+^RGCs per retina. Data are presented as mean ± SD, with statistical significance assessed using one-way ANOVA with Tukey’s post hoc test for multiple comparisons. LPS, lipopolysaccharide.

To evaluate whether LPS inoculation affect directly RGC well-being, we first examined the Brn3a appearance in retinas immunodetected with Iba 1 (Figure 2A). Brn3a signal qualitatively appears similar among groups suggesting that the LPS induced inflammation does not cause RGC to have any pathological state, since Brn3a is a marker for RGC survival but also indicates the healthy state of RGCs [56]. To further investigate whether the MC shift towards an activated phenotype induced by LPS observed by their morphology (Figure 1D) could induce RGC degeneration, we quantified the total number of RGCs. First we also confirmed by manual quantification the microglia cell density increases in a representative retina (Figure 2B,D first row). At early time points (3, 6, and 24 h), the inoculation of LPS did not provoke significant increase in cell number (Figure 2B) and the distribution in the retinal surface remains evenly and similar to control (Figure 2D, first row), and to previous studies [11]. However, The MCs at later stages showed a denser MC population of hypertrophic cell bodies with wider processes and exhibiting migration toward retinal blood vessels (Figure 2A, see merged images). Consistent with this observation the k-neighbor map at 9d after inoculation, shows denser MC population showing diffusely/broadly distributed along the whole retina. Second, the appearance of RGC in retinas after LPS administration does not differ from those intact retinas (Figure 2A, second row). Quantification of total RGC numbers in whole-mounted retinas across all experimental groups revealed that systemic LPS administration did not induce changes in total RGC numbers at any time point examined (Figure 2C). Examining the topography of Brn3a^+^RGCs demonstrated absence of any regional or diffuse RGC loss (Figure 2D, second row).

### 2.3. LPS-Induced Systemic Inflammation Increases the RGC Susceptibility to ONC Degeneration

Systemic inflammation induced by LPS inoculation caused MC activation without RGC loss. Thus, we hypostatized that LPS-induced systemic inflammation activated retina MC could exacerbate RGC loss following ON injury.

**Figure 3:**
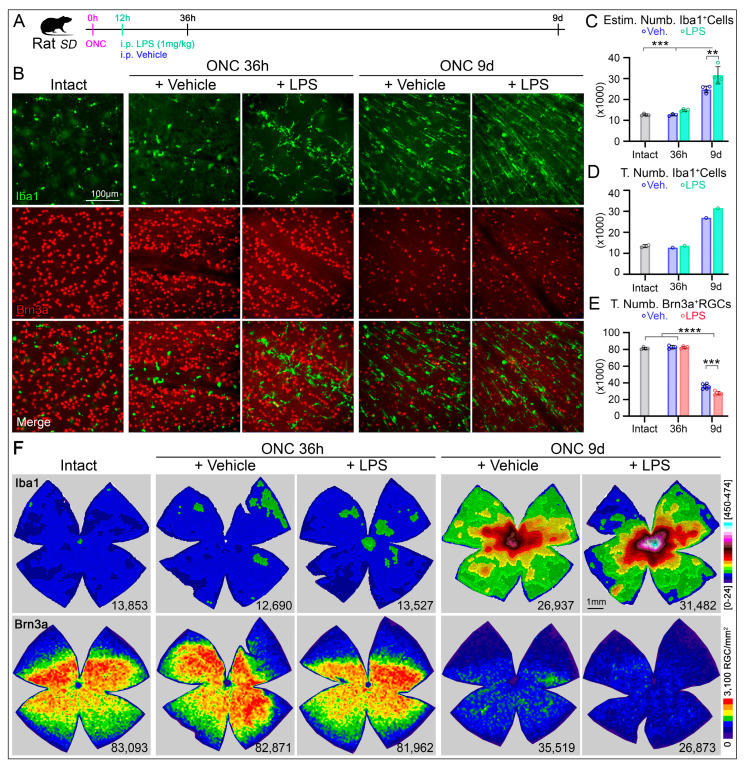
Exacerbated RGC loss following lipopolysaccharide inoculation after optic nerve (ON) injury. (**A**) Experimental design, the effects of intraperitoneal (i.p.) administration of LPS on the RGC survival after optic nerve crush (ONC). (**B**) Representative images of microglial cells (Iba1^+^, green) and RGCs (Brn3a^+^, red) in intact retinas or after different times post-ONC and i.p administration of LPS or vehicle. (**C**) Bar graphs show significantly higher estimated number of microglial cells at 9 days after ONC. I.p. administration of LPS significatively increases the numbers of microglial cells at 9 days post-ONC (sample sizes: Intact, n = 4; ONC Veh. 36 h, n = 3; ONC LPS 36 h, n = 4; ONC Veh 9d, n = 4; ONC LPS 9d, n = 4). (**D**) Bar plots from quantification in whole retinas (n = 1 retina/group; except to Intact group, n = 2) confirm the estimated numbers (**B**). (**E**) Bar graphs showing a significant Brn3a^+^RGCs loss after ONC. I.p. administration of LPS significatively increase the RGC loss at 9 days post-ONC (sample sizes: Intact, n = 4; ONC Veh. 36 h, n = 3; ONC LPS 36 h, n = 4; ONC Veh 9d, n = 6; ONC LPS 9d, n = 5). (**F**) K-nearest neighbor maps showing the topographical distribution of Iba1^+^cells (top row) and Brn3a^+^RGCs (bottom row) in rat retinas. The retinas at 9d post ONC exhibit higher Iba1^+^cell density, that is more pronounced after LPS administration. RGC densities show a massive RGC loss at 9d post ONC that was greater in the LPS group. Color scales (left) indicate local density ranging from [0–24 neighbors/cell] (purple) to [450–474 neighbors/cell] (red) or (cyan) within a 345 µm radius. The bottom right of each map indicates the total number of Iba1^+^cells or Brn3a^+^RGCs per retina. Data are presented as mean ± SD, differences between ONC groups (vehicle vs. LPS) were assessed using two-way ANOVA with Tukey’s post hoc test for multiple comparisons. Comparisons between each ONC group and the intact group were performed using one-way ANOVA with Tukey’s post hoc test. Statistical significance is indicated as follows: *p* < 0.01 (**), *p* < 0.001 (***) and *p* < 0.0001 (****). LPS, lipopolysaccharide.

After ONC, we administered a single injection of LPS or vehicle at 12 h. The retinas were analyzed in 2 phases of the RGC degeneration after ONC (Figure 3A): at 36 h during the early acute phase that will corresponds to 24 h after the i.p. injection, time enough to induce at least two significative changes (the skeletonized length and soma size, Figure 1E,G); and at 9d, in the later acute phase when most of the RGC have degenerated [11,17], but with the additional increased number of MCs observed in intact retinas (Figure 2D). Anatomically, double staining with Brn3a and Iba1 demonstrate morphological MC changes after ONC at 36 h (Figure 3B, first row), but no RGC loss (Figure 3B, second row). The administration of LPS, in addition causes a slight migration of MCs towards the vasculature (Figure 3B, first row). At 9 days post-ONC, microglial cell density increases in both groups, but with a significant increase in the LPS treated group towards the ON head (ONH). In relation with RGC survival, both groups show a significant loss of RGCs (left panels in Figure 3B).

Total numbers of MCs were not different between intact retinas and ONC injured retinas with intraperitoneal administration of vehicle or LPS examined at 36 h (Figure 3C,D, Table 2). However, at 9d post-ONC (and 8.5 days after LPS inoculation) we observed a significant increase of MC numbers either after estimating the total number (31,631 ± 4123, Figure 3C) or after manual quantification (31,482, Figure 3D) compared to the vehicle treated group (24,843 ± 1596 and 26,937, respectively, Figure 3C,D, Table 2). Interestingly, when we quantified the total number of RGCs, we did not observe significant differences at 36 h post-ONC for the LPS and vehicle treated groups (82,573 ± 1216 vs. 82,758 ± 2140, respectively; Figure 3E, Table 2). Although the systemic inflammation induced by LPS inoculation did not induce a significant reduction of RGCs in naïve SD rats (Figure 2C,D, bottom row), the same LPS dose reduced by an additional 22% the number of RGCs in retinas at 9 days after ONC (35,645 ± 2760, Figure 3E, Table 2) compared to the Vehicle at 9d post ONC (27,649 ± 2091, Figure 3E, Table 2).

Observing the spatial distribution, the microglia cell topographies at 36 h post ONC do not differ significantly between both groups, except for the central retinal area surrounding the ONH (Figure 3F, middle columns). At 9d post-ONC the accumulation of the microglia after LPS significantly changes, being more dramatically localized towards the ONH (Figure 3F, left columns). The isodensity maps for Brn3a^+^RGCs at 36 h post-ONC demonstrate similarities between LPS or vehicle administration (Figure 3F, middle columns) when compared to the intact retinas (Figure 3F, left column). However, at 9d post-ONC the loss of RGC was exacerbated after LPS administration compared to the vehicle treated group (Figure 3F, left columns). These maps in concordance with quantitative data indicate that LPS-activated microglial cells can exacerbate the RGC degeneration during the late, but not in the early phase of axotomy induced RGC loss.

### 2.4. Different Microglial Cell Profiles After LPS-Induced Systemic Inflammation in Intact and Injured Retinas

**Figure 4:**
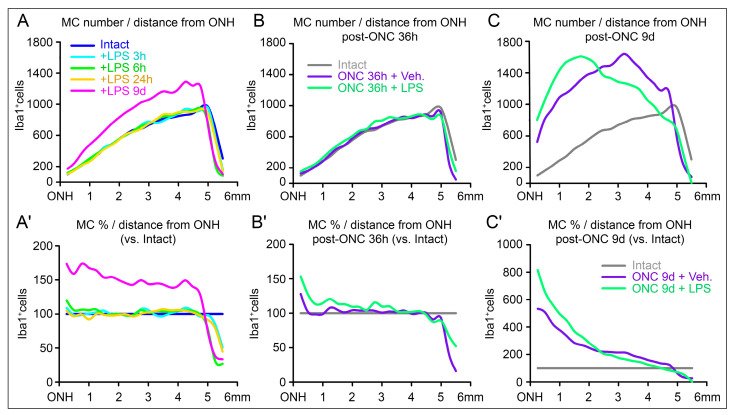
Microglial dynamics after lipopolysaccharide inoculation and ON injury. Cumulative microglial cell profiles from the ON head (ONH). Line graphs where the mean number or percentage of cells (Y-axis) counted after LPS inoculation are plotted against the length of the retina (X-axis) being 0 the ONH and 6 mm the periphery. (**A**–**C**) Profiles of number of Iba1^+^MCs against distance from ONH. (**A′**) Iba1^+^MCs as percent of intact retinas (being 100% the number of SMCs in intact retinas, blue line). (**B′**,**C′**) Iba1^+^MCs as percent of ONC at 36 h or 9d respect intact (100%, gray lines). Left column shows number and percentage of intact and control retinas at different times post-LPS inoculation. Center and right column show the direct comparison of the MCs numbers and percentages after ONC and at 36 h or 9d post-LPS inoculation.

These regional aggregation of MCs exemplified by comparing the k-neighbor map of MCs in the whole retina can be simplified as the MC profiles versus the distance from the ONH. In intact retinas only subjected to the LPS inoculation, the MCs increased only to 9 days along the entire retinal length (Figure 4A). Relative comparison versus the percentage in intact retinas demonstrate a slight increase towards the central retina (towards the ONH, Figure 4A′). Following LPS injection and ONC, at 36 h the MC profiles are almost identical after administering LPS or vehicle (Figure 4B), and only a minor difference with more proportion of cells towards the ONH is detected in the proportion or relative profiles (Figure 4B′). Interestingly, when we compare the density of MCs between ONC with LPS or vehicle administration, the profiles are different. After LPS, the MC trend accumulates towards the central retina, while they are preferentially located towards mid periphery after vehicle administration (Figure 4C). These differences are clearly visible in the relative profiles consistently demonstrating higher aggregation of MC in the central retina near the ONH after LPS administration (Figure 4C′). Thus, in both analyzed time points the LPS administration induces a tendency to aggregate MCs towards the ONH.

## 3. Discussion

In the present study, we investigated how systemic inflammation induced by LPS shapes retinal microglial responses and RGC vulnerability under physiological conditions on following ONC. Our data demonstrates that systemic LPS administration rapidly activates retinal microglia, inducing pronounced morphological changes within hours, yet this activation alone is insufficient to trigger RGC degeneration in intact retinas. Importantly, when combined with axonal injury, the same inflammatory stimulus significantly exacerbates RGC loss during the late acute phase of degeneration, accompanied by a marked increase and central redistribution of microglial cells toward the ONH. These findings highlight the context-dependent effects of microglial activation and support the idea that systemic inflammation acts as a priming factor that aggravates neuronal loss only when neuronal integrity is already compromised.

### 3.1. Rapid Microglial Activation Following Systemic LPS Without Early Changes in Cell Number

Although microglia are often described as “resting” under physiological conditions, they are highly dynamic cells that continuously survey the neural parenchyma [2,57]. Detection of non-physiological signals rapidly initiates inflammatory responses [6], particularly through pathogen-associated recognition receptors such as Toll-like receptors (TLRs; [30,31,50,58]), which trigger defense neuroinflammatory programs [59,60,61]. Consistent with this, systemic LPS administration induced a rapid microglial response in the retina, characterized by process retraction, soma remodeling, and a transition from a highly ramified to a fusiform or rod-like morphology as early as 3 h post-injection.

These morphological changes occurred in the absence of detectable increases in microglial cell number, indicating that systemic inflammation initially alters microglial activation state rather than inducing immediate proliferation or recruitment. Quantitative analyses confirmed reduced branching complexity and progressive hypertrophy, consistent with an activated phenotype [62] as previously described [63,64]. Although increased microglial density was detected at later time point (9d), proliferation or recruitment may occur earlier than assessed here, as reported in other CNS regions (after 48 h in brain [65]. Nevertheless, these data demonstrate that retinal microglia rapidly respond to systemic inflammatory cues independently of early changes in cell number.

### 3.2. LPS-Induced Microglial Activation Is Not Sufficient to Induce RGC Loss in Intact Retinas

Systemic LPS administration has been associated with widespread physiological and neurological alterations, including metabolic dysfunction, suppressed locomotor activity and weight loss in mice [66], depression-like behaviors and memory deficit [67], cognitive impairment associated with hippocampal neurodegeneration [68], and loss of dopaminergic neurons in the substantia nigra of adult mice [38]. In the visual system, LPS could be associated with different retinal degenerations [47,48,49] and it was recently reported RGC loss in mice following systemic LPS exposure, with increased susceptibility in males [39], and inducing demyelination and axonal and RGC loss when locally applied to the ON [54].

In contrast, despite robust microglial activation, systemic LPS administration in rats did not induce RGC loss or alter the spatial distribution of Brn3a-positive RGCs at any time point examined. This dissociation highlights a critical distinction between microglial activation and microglia-mediated neurotoxicity. Although activated microglia can release pro-inflammatory cytokines, reactive oxygen species, and complement factors capable of damaging neurons [69,70,71,72,73], such effects likely require additional permissive conditions, including pre-existing neuronal stress or injury.

Similar dissociations between inflammation and neuronal loss have been reported in other rat models, where inflammatory activation alone induced learning and memory deficits without overt neuronal degeneration [37]. Moreover, in degeneration models, microglial activation by itself was insufficient to induce neuronal degeneration but significantly increased neuronal susceptibility under pathological conditions (such as enhancing photoreceptor degeneration in models of retinitis pigmentosa following LPS administration [53], or facilitating the development of cerebral edema during short hypoxia [74]. Together, these findings suggest species-specific differences and indicate that, in the intact rat retina, LPS may undergo functional changes without triggering neuronal degeneration or, that potentially harmful microglial effects may be counterbalanced by protective mechanisms and regulatory interactions with other retinal glial cells [75].

### 3.3. Systemic Inflammation Primes the Injured Retina and Exacerbates Late-Phase RGC Degeneration

Similar to systemic LPS inoculation, neuronal injury initiates a robust neuroinflammatory response [70] driven by the release of endogenous danger signals such as ATP, HMGB1, and nucleic acids, which activate microglia through receptors including TLRs, P2X7R, RAGE, and GPR84 [76,77,78,79,80]. These pathways also promote inflammatory transcriptional programs and pro-inflammatory microglial polarization [81], accompanied by rapid cytokine release following trauma [82]. Consistent with these mechanisms, microglia rapidly exhibit classical activation (cell body enlargement, process retraction, transformation toward an amoeboid morphology; [62]), together with the release of pro-inflammatory mediators [83], migration toward injury sites and increased phagocytic activity accompanied by the progressive RGC degeneration [9,11,17,22,23,24].

A key finding of the present study is that systemic LPS-induced inflammation exacerbates RGC degeneration after ONC in vivo, but selectively during the late acute phase (recently associated to neuroinflammation; [84]). At 36 h post-injury, when early apoptotic signaling is already underway, LPS administration did not further reduce RGC survival. In contrast, at 9 days post-ONC (when the majority of RGC degeneration has occurred) LPS-treated animals exhibited a substantial additional loss of RGCs compared to vehicle-treated controls. This temporal dependency suggests that systemic inflammation does not accelerate the initial wave of injury-induced apoptosis signaling but instead amplifies secondary degenerative mechanisms, including sustained microglial activation, prolonged cytokine release, complement engagement, and disruption of neuron–glia homeostasis. Similar effects of systemic immune challenges support this interpretation, as exacerbating neuronal loss following ischemia or traumatic brain injury by amplifying local inflammatory cascades [85,86,87].

Importantly, it is currently believed that retinal microglia are not directly exposed to circulating LPS; rather, systemic immune activation induces inflammation at a distance. Accordingly, early retinal microglial activation is thought to occur indirectly, mediated by circulating cytokines [88], endothelial signaling, or alterations in blood–retina barrier permeability [89]. This indirect mode of activation may lead to a qualitatively distinct microglial phenotype compared to microglia directly stimulated by LPS in vitro or ex vivo. Supporting this idea, exposure of organotypic mouse retinal explants to LPS did not induce RGC loss for up to 14 days post-explantation [90], or even promoted RGC survival [91]. Together, these observations may explain why systemic LPS administration alone is insufficient to induce retinal degeneration yet becomes deleterious when combined with axonal injury. Systemic inflammation is often associated with responses of greater magnitude and longer duration, which are linked to worse neurological outcomes [92], with the complement system playing a key role in pathological neuronal elimination [93]. Interactions with other retinal glial cells further amplify these effects, as astrocytes can adopt cytotoxic phenotypes [94,95] and Müller cells can secrete pro-inflammatory mediators that promote RGC apoptosis [96].

In addition, microglial phagocytic activity is additionally regulated by “eat-me” signals, such as phosphatidylserine externalization, which activate TLR-dependent pathways [97,98,99]. Notably, microglial activation can also be induced independently of overt degeneration, as demonstrated by intravitreal injection of apoptotic neurons [100], or amplified through microglia-derived exosomes generated under pathological conditions [101]. These mechanisms support the concept of a self-amplifying inflammatory state that promotes secondary neuronal apoptosis, even in the absence of direct neuronal injury.

### 3.4. Central Redistribution of Microglia Toward the ONH as a Site of Vulnerability

Topographic analyses revealed that systemic LPS promotes microglial accumulation in the central retina and around the ONH following ONC, a redistribution that emerged at both analyzed degenerative phases, but more significatively at 9d post-ONC coinciding with RGC loss. Systemic LPS induce microglial migration [102], and our findings indicate that this migratory response may be preferentially directed toward the ONH. The ONH represents a region of high axonal density, metabolic demand, and susceptibility to mechanical and ischemic stress. Microglia interact closely with the retinal vasculature, regulating endothelial function and barrier integrity [103], effects that are also observed following systemic LPS exposure [32,33,34]. Enhanced microglial accumulation near the ONH may therefore reflect regional vulnerability to inflammatory amplification, facilitating sustained cytokine signaling, complement activation, and prolonged neuroinflammatory responses. In the context of combined systemic inflammation and axonal injury, vascular dysfunction at the ONH may further amplify local inflammatory signaling and could potentially favor increased immune cell trafficking into the tissue, thereby contributing to the heightened vulnerability of this region.

### 3.5. Dual and Context-Dependent Roles of Microglia in Retinal Degeneration

Microglia exhibit marked functional plasticity, and their roles in neurodegeneration are highly context dependent. Activated microglia can promote neuronal survival through neurotrophic and anti-inflammatory signaling, yet sustained activation can drive degeneration through chronic release of pro-inflammatory mediators such as TNF-α, IL-1β, and IL-6 [104,105,106]. The balance between these opposing roles appears to depend on the nature, intensity, and duration of the activating stimulus, as well as the vulnerability state of the affected neurons. Our findings support a model in which systemic inflammation primes retinal microglia toward a heightened reactive state that is relatively non-deleterious in intact tissue but becomes harmful when combined with injury-induced signals. It is possible that under such conditions, microglia may engage in self-perpetuating inflammatory loops involving astrocytes and Müller cells, amplifying neurotoxic signaling and impairing tissue recovery [96]. This interpretation aligns with transcriptomic studies revealing multiple microglial activation states beyond the classical M1/M2 dichotomy [107,108]. Activated microglia can themselves release a combination of pro-inflammatory cytokines that interact synergistically with neighboring glial cells, reinforcing a degenerative microenvironment [104]. Among these mediators, TNF has been shown to initiate extrinsic apoptotic pathways in neurons [105], IL-1β can exert direct neurotoxic effects while simultaneously activating astrocytes [109], and IL-6 has been implicated in sustaining chronic neuroinflammatory responses [106]. Together, these signaling cascades may maintain microglia in a prolonged reactive state that favors secondary degeneration rather than resolution and repair.

### 3.6. Therapeutic Implications, Limitations, and Future Directions

Traumatic neuronal injury in the CNS often leads to long-term disability due to the limited regenerative capacity of mature neurons [25]. Early functional deficits frequently precede progressive degeneration, highlighting the importance of timely intervention. Our data reinforces the concept that therapeutic strategies should aim to modulate, rather than eliminate, microglial responses following ON injury. Indeed, microglial depletion does not improve RGC survival after ONC [28], whereas selective attenuation of inflammation using non-steroidal anti-inflammatory drugs reduces RGC loss [110,111,112,113]. Importantly, our findings further indicate that systemic inflammatory status critically influences retinal outcomes and should be considered when designing neuroprotective strategies. Potential therapeutic approaches include agents that limit excessive microglial toxicity [114,115], promote regulatory activation states [116], regulate autophagy [117], or exert combined anti-inflammatory and endothelial-protective effects [118,119]. Timing is likely to be critical, as early modulation may interfere with beneficial inflammatory responses, whereas late-phase intervention may prevent chronic neurotoxicity.

Several limitations should be acknowledged. We did not assess functional visual outcomes or directly examine interactions between microglia and other retinal glial populations. Although our analyses demonstrate that systemic LPS exacerbates microglial activation and increases RGC loss following optic nerve crush, functional consequences on RGC activity were not directly evaluated. Given the established impact of neuroinflammation on synaptic integrity, axonal conduction, and neuronal excitability, it is likely that visual function would be impaired not only due to increased RGC loss, but also due to functional deficits in surviving neurons. Such dysfunction may precede or exceed structural degeneration, particularly in conditions of systemic inflammation. Accordingly, functional alterations may be especially relevant in intact retinas exposed to systemic LPS, where the absence of overt RGC loss does not preclude altered neuronal function. From a translational perspective, systemic inflammatory states such as sepsis could therefore predispose patients to transient or permanent visual impairment, an effect that may be exacerbated when sepsis coincides with facial trauma involving the retina or optic nerve. Future studies incorporating electrophysiological [39,120], behavioral [121,122], and cell-type-specific approaches [123] will be essential to fully define the functional consequences of systemic inflammation on retinal neurodegeneration.

Another limitation of the present study is that whole-retina microglial numbers were primarily estimated from regional density measurements rather than exhaustive manual counts of all retinas. Although this approach is widely used and regional densities were sampled across multiple eccentricities, it may not fully capture subtle inter-animal variability or fine-grained spatial heterogeneity. To partially address this, full-retina topographical maps were generated from representative retinas whose estimated microglial densities closely matched group averages, and these showed distributions consistent with those observed across animals within each group. Nevertheless, future studies incorporating a larger number of fully quantified retinas would further strengthen confidence in absolute microglial counts and spatial distribution analyses. It should be noted that naïve animals, rather than contralateral eyes, were used as controls for ONC experiments. Although contralateral eyes are often employed as internal controls, accumulating evidence indicates that unilateral damage to the optic nerve or the retina can induce bilateral glial responses, potentially confounding interpretation. To minimize such effects, we opted to use naïve retinas as controls; however, this approach precludes paired intra-animal comparisons.

Additionally, the association between enhanced microglial activation and exacerbated retinal ganglion cell loss following optic nerve injury under systemic LPS exposure remains correlative. Although the spatial redistribution and activation of microglia strongly support a contributory role in the observed neurodegeneration, this study did not include direct experimental manipulation of microglial activity, such as pharmacological depletion, inhibition, or phenotypic modulation, nor did it establish a definitive molecular link through specific inflammatory mediators. Therefore, causal inferences regarding microglia-mediated neurotoxicity should be made with caution. Future studies employing targeted microglial interventions and mechanistic analyses of inflammatory signaling pathways will be necessary to determine the extent to which microglia actively drives neuronal loss in this context.

## 4. Materials and Methods

### 4.1. Animal Handling

Adult female Sprague-Dawley rats weighing ~200 g (2-month-old, n = 42) were bred in the breeding colony of the University of Murcia and housed under light- and temperature-controlled conditions (21–23 °C) with free access to food and water. This study was conducted in accordance with the European Union and Spanish guidelines for Animal Care and Use for Scientific Purpose (Directive 2010/63/EU and Royal Decree 53/2013, respectively), and the Association for Research in Vision and Ophthalmology (ARVO) Statement for the Use of Animals in Ophthalmic and Vision Research. All of the protocols were approved by the Ethical and Animal Studies Committee of the University of Murcia and the Biomedical Research Institute of Murcia (IMIB), Spain (REGA 300305440012).

Anesthesia was induced by intraperitoneal injection of a mixture of ketamine (60 mg/kg, Imagene^®^, Alcobendas, Madrid, Spain) and xylazine (10 mg/kg, Rompun^®^, Bayer, Kiel, Germany). Analgesia was provided by subcutaneous administration of buprenorphine (0.1 mg/kg; Buprex, buprenorphine 0.3 mg/mL; Schering-Plough, Madrid, Spain). During surgery, the eyes were covered with a topical ointment (Tobrex; Alcon, S. A., Barcelona, Spain) to prevent corneal desiccation.

### 4.2. Experimental Design

Thus, the animals were clustered into the following groups: (i) intact, without LPS neither surgery; (ii) LPS-induced systemic inflammation, analyzed 3-, 6- and 24-h, and 9-days after a single i.p. administration of LPS; (iii) Optic nerve crush (ONC) with a single intraperitoneal injection of LPS administered 12 h post-injury, with retinal analyses performed at 36 h and 9 days after ONC; and (iv) ONC with vehicle (sterile isotonic saline) administered 12 h post-injury, analyzed at the same time points. A schematic representation of the experimental design is shown in Figure 1A and Figure 3A. The selected time points were based on previous studies demonstrating that systemic LPS induces a neuroinflammatory response within approximately 3–6 h after administration [63,64,124], and to capture both early and delayed inflammatory and neurodegenerative events.

### 4.3. Lipopolysaccharide Preparation and Administration

To induce the systemic inflammation mimicking a septic shock [125,126], the epithelial barrier that protects from invading microorganisms was leapt by injecting intraperitoneally 0.2 mL of a solution containing 1 mg/mL purified Lipopolysaccharide (LPS) -endotoxin from gram-negative bacteria- (LPS25 *E. coli* O111:B4, Sigma, St. Louis, MO, USA) in free sterile isotonic saline. This dose (1 mg/kg) was chosen for its consistence on inducing endotoxemia and innate immune cells activation through Toll-like receptor-4 (TLR4) [39,127] and it was administrated at the same time of the day (approx. 10.00 am). The single inoculation of LPS will cause sepsis and the body will become overactive in response to the infection. During the course of the experiment, sickness-related behaviors (body weight, body temperature, and reductions in movement and grooming) were monitored to confirm successful induction and to minimize distress in the experimental animals. In addition, for three consecutive days, animals received subcutaneous injections of saline solution (250 µL) to ensure adequate hydration.

### 4.4. Optic Nerve Crush Procedure

To induce the ON trauma and trigger RGC degeneration, the left ON was crushed, for 10 s, at 2.5–3 mm from the optic disc as previously reported [17,21,24,56]. Although ONC does not induce contralateral degeneration of RGCs in rats [24], contralateral eyes were not used as controls in the present study because systemic LPS administration may elicit a retinal inflammatory response. Instead, retinas from intact animals were used as controls.

### 4.5. Tissue Preparation

Animals were sacrificed by an intraperitoneal administration of an overdose of sodium pentobarbital (Dolethal, Vetoquinol; Especialidades Veterinarias, S.A., Alcobendas, Madrid, Spain). All animals were perfused transcardially with 0.9% saline solution followed by 4% paraformaldehyde in 0.1 M phosphate buffer. Later, eyes were enucleated, and retinas were gently dissected as flattened whole mounts by four relaxing cuts, the deepest oriented towards the dorsal retina as previously described [11,17,20,24,56]. Fixed tissues were safely stored in cold Phosphate-Buffered Saline (PBS, 4 °C) with sodium azide (0.1%) for short periods, before subsequent staining steps.

### 4.6. Immunohistochemistry

Double immunodetection of RGCs and MCs in retinal flat mounts was conducted as previously described [11]. Immunohistochemical staining with primary antibodies to Brn3a (C-20; Santa-Cruz Biotechnology, Heidelberg, Germany) diluted 1:750, a marker exclusively expressed by RGCs [17,128]; and to ionized calcium-binding adaptor molecule-1 (Iba1; Wako Chemicals GmbH, Neuss, Germany) diluted 1:1000, a marker constitutively expressed by microglia [129] were performed. Whole-mount retinas were washed with 0.5% Triton-100x in PBS and incubated with the primary antibodies overnight at 4 °C. Thereafter, retinas were washed with the same solution and were incubated for 4 h at room temperature with the secondary antibodies diluted at 1:500 (Alexa Fluor 594 and 488 respectively; Molecular Probes; Thermo Fisher Scientific, Madrid, Spain). Finally, the retinas were mounted with the GCL facing up and cover-slipped with antifade mounting media.

### 4.7. Image Acquisition

Whole-retinal photomontages were acquired using an epifluorescence microscope (Axioscop 2 Plus; Zeiss Mikroskopie, Jena, Germany) equipped with a computer-driven motorized stage (ProScan H128 Series; Prior Scientific Instruments, Cambridge, UK) controlled by image analysis software (Image-Pro Plus, IPP 5.1 for Windows; Media Cybernetics, Silver Spring, MD, USA). Retinal photomontages focused on the GCL were reconstructed from 11 × 14 individual 10× images by zigzag tiling, as reported [11,17,20].

### 4.8. Quantification

RGCs (Brn3a^+^nuclei) in all retinas were automatically quantified using a previously developed algorithm (FIJI-ImageJ 1.54p, NIH, Bethesda, MD, USA; [19]). MCs (Iba1^+^) were manually estimated in the whole retina due to their variant morphology that impossibilities an accurate automated quantification. Briefly, by a supervised algorithm [11,130], we extract individualized images (0.25 mm^2^/sample) into the four retinal quadrants at three concentric areas (central, equatorial, and peripheral). Thus, four images per ring were manually dotted (Adobe Photoshop 21.2.4; Adobe Systems, Inc., San Jose, CA, USA), and its averaged number was used to calculate the density per each concentric area. Thus, the MC density per retina was the sum of these partial values. Finally, the total number per retina was estimated by multiplying the average density (cells/mm^2^) by their corresponding total retinal area (in mm^2^). In addition, to further corroborate our MC number estimation and visually assess their spatial distribution along the retina, a representative retina per group was manually dotted using an editing software (Photoshop 21.2.4) to obtain the total number and *x*,*y* coordinates of each MC, as previously described [11,19]. Quantitative data from all populations were exported to a spreadsheet application (Microsoft Office Excel 365; Microsoft Corporation, Redmond, WA, USA) for further analysis.

### 4.9. Iba-1 Positive Cells Morphology

Microglial response was evaluated in individualized images selected from the four quadrants of the retinal photomontages (10×) with Iba-1 immunostaining. Briefly, brightness/contrast was adjusted to better visualize the faint microglia processes. The cell body and processes were then manually skeletonized using the brush tool (white, 1 pix thickness) of an editing software (Photoshop 21.2.4). The images were converted to binary by adding a new black layer and used to quantify automatically the total length and manually the number of branch points (single pixel dot, Photoshop 21.2.4). Additionally, the original images were used to outline the cell soma using the lasso tool (Photoshop 21.2.4) as previously described [131]. Then the individualized processed images from a single cell (3 images/cell, total length, branch points and soma size) were used to quantify automatically total number of white pixels using the threshold tool (FIJI-ImageJ 1.54p) as previously described [98]. The sample sizes analyzed were 24 cells from n = 4 retinas for intact animals, 30 cells from n = 4 retinas for 3 h-LPS treated animals, 31 cells from n = 4 retinas for 6 h-LPS treated animals, 38 cells from n = 5 retinas for 24 h-LPS treated animals, 21 cells from n = 3 retinas for 9d-LPS treated animals.

### 4.10. Spatial Distribution

Detailed spatial distribution of Brn3a^+^RGCs was visualized by isodensity maps using scientific graphing software (Sigma Plot 11.0 for Windows; Systat Software, Inc., Richmond, CA, USA). Isodensity maps are filled with contour plots depicting the RGC topography in 3 dimensions using x,y coordinates and a color code for the cellular density, from 0 (purple) to 3100 (red) RGCs/mm^2^, as previously reported [17,56]. The Iba1^+^MC topography, instead, was represented by k-neighbors maps [11]. Briefly, the position of each dot (representing an Iba1^+^MC) in the photomontage was translated to an *x*,*y* location using reference the ONH center (0,0 point). The k-neighbour algorithm (Java SE8 Update 541, ORACLE Corporation, Redwood Shores, CA, USA) calculated the number of cells in the vicinity of each cell within a given radio (345 µm). Plotted Maps using same scientific graphing software (Sigma Plot 11.0) depict the number of neighbors around using a color code, purple [0–24 neighbors] to white [450–474 neighbors]. Additionally, processed data was used for analyzing the distribution profiles as previously performed [11,24].

### 4.11. Statistical Analysis

Statistical analyses among groups were performed using one- and two-way analyses of variance (ANOVA), and multiple comparisons between pairs of group means performed with post hoc Tukey’s test (GraphPad Prism v.10, GraphPad San Diego, CA, USA). Normality of data distribution was assessed using the Shapiro–Wilk test, and homogeneity of variances was evaluated using Levene’s test. Data are shown as mean ± standard deviation (SD) and differences were considered significant at *p* < 0.05 as the basis for rejecting the null hypothesis. Specific analyses are detailed in the corresponding figure legends.

## 5. Conclusions

In summary, this study demonstrates that systemic LPS-induced inflammation rapidly activates retinal microglia without causing RGC loss in intact retinas but significantly exacerbates RGC degeneration following ON injury. This deleterious effect emerges during the late acute phase and is associated with increased microglial accumulation in the central retina and around the ONH. These findings support the concept that peripheral inflammation amplifies the neurodegenerative response to CNS trauma through microglial activation, emphasizing the systemic immune state as a determinant of retinal and CNS injury outcomes. Targeted, time-dependent modulation of microglial responses may therefore represent a promising strategy for neuroprotection in optic neuropathies.

## Figures and Tables

**Table 1:** Total number of MC and RGC, and skeletonized results in intact and at different time points after LPS administration.

		Intact	+LPS 3 h	+LPS 6 h	+LPS 24 h	+LPS 9d
**MC Number**	**Estimated**	12,641 ± 473	12,926 ± 424	12,835 ± 408	13,333 ± 251	19,071 ± 1051
	**Quantified**	13,355 ± 704	13,204	12,709	13,073	17,599
**RGC number**	**Quantified**	81,355 ± 1249	81,865 ± 2173	82,177 ± 2921	82,491 ± 2810	81,832 ± 3623
**Skeleton**	**Length (pix)**	340 ± 25 (100%)	122 ± 19 (−64%)	125 ± 16 (−63%)	135 ± 30 (−60%)	113 ± 6 (−67%)
	**Nodes**	22.5 ± 1.4 (100%)	5.8 ± 1.1 (−74%)	6.3 ± 1.6 (−72%)	6.1 ± 1.7 (−73%)	6.4 ± 1 (−72%)
	**Soma (pix)**	48.2 ± 2.7 (100%)	39.4 ± 4.7 (−18%)	43.6 ± 8.9 (−10%)	70.2 ± 10.7 (+45%)	107.9 ± 30.8 (+124%)

Note for the microglial cell (MC) total number by quantification the sample size was 1 for all groups except for the Intact group (n = 2). For skeletonized length, nodes and soma in parenthesis is shown the percentage of reduction (negative %) or the increase (positive %) considering 100% the values for intact retinas.

**Table 2:** Total number of MC and RGC, at different time points after ONC and LPS administration.

		Intact	ONC 36 h		ONC 9d	
			+Vehicle	+LPS	+Vehicle	+LPS
**MC Number**	**Estimated**	12,641 ± 473	12,670 ± 557	14,929 ± 498	24,843 ± 1596	31,631 ± 4123
	**Quantified**	13,355 ± 704	12,690	13,527	26,937	31,482
**RGC number**	**Quantified**	81,355 ± 1249	82,758 ± 2140	82,573 ± 1216	35,645 ± 2760	27,649 ± 2091

Total number of MC and RGC, at different time points after ONC and LPS administration. Note for the MC total number by quantification the sample size was 1 for all groups except for the Intact group (n = 2). RGCs were quantified automatically.

## Data Availability

All data generated or analyzed during this study is available from the corresponding author upon reasonable request.

## Abbreviations

The following abbreviations are used in this manuscript:

Brn3a	POU4F1, a class IV POU domain-containing transcription factor
d	days
GCL	Ganglion Cell Layer
h	hours
MCs	Microglial cells
Iba1	Ionized calcium-binding adaptor molecule 1
LPS	Lipopolysaccharide.
ON	Optic nerve
ONH	Optic nerve head
ONC	Optic nerve crush
PBS	Phosphate-Buffered Saline
RGCs	Retinal ganglion cells
SD	Sprague-Dawley rat
Veh.	Vehicle
